# Description of the first two cases of Pompe disease in Bulgaria

**DOI:** 10.1186/1471-2474-14-S2-P21

**Published:** 2013-05-29

**Authors:** I Tournev, T Chamova, I Sinigerska, V Guergueltcheva, M Gospodinova, P Ganova, R Stoilov, A Todorova, T Todorov

**Affiliations:** 1 Clinic of Neurology, University hospital “Alexandrovska”, Sofia, Bulgaria; 2 Department of Cognitive Science and Psychology, New Bulgarian University, Sofia, Bulgaria; 3 National Genetics Laboratory, Medical University, Sofia, Bulgaria; 4 Clinic of cardiology, University hospital “Alexandrovska”, Sofia, Bulgaria; 5 Clinic of rheumatology, University hospital “Ivan Rilski”, Sofia, Bulgaria; 6 Genetic Medico-Diagnostic Laboratory “Genica”, Sofia, Bulgaria; 7 Department of Medical Chemistry and Biochemistry, Sofia Medical University, Sofia, Bulgaria

## Introduction

Pompe disease is a rare, metabolic, multi-system, lysosomal storage disorder with autosomal recessive inheritance, caused by a deficiency of the glycogen-degrading lysosomal enzyme, acid alpha-glucosidase (GAA). Great phenotypic variability has led to the classification of several subtypes: infantile, late-infantile, childhood, juvenile, and adult-onset form, based on the age of onset and degree of organ involvement. In the most severe cases, disease onset is in infancy and death results from cardiac and respiratory failure along with muscle weakness within the first one or two years of life. In the milder, late-onset forms, muscle weakness is the primary symptom. Weakness of respiratory muscles is the major cause of mortality in these cases.

## Results

We present the first two cases of Pompe disease in Bulgaria. The first patient is a 57-year-old female, with onset of the disease at the age of 54, and a slow progression of limb-girdle muscle weakness, restrictive-obstructive type of respiratory weakness, and liver involvement. The second patient is a 45-year-old male with clinical onset at the age of 35 with proximal muscle weakness in the lower limbs and restrictive respiratory weakness. The activity of acid alpha glucosidase in dried blood spot samples was markedly reduced and subsequently the genetic testing proved that both patients carried the same mutations as double heterozygous for g.-32-13T>G in intron 1 and c.1655T>C; p.(Leu552Pro) in exon 12 of the GAA gene. The first mutation is quite common in Caucasians, while the second one is described only in Greek patients.

## Conclusion

We can speculate that c.1655T>C; p.(Leu552Pro) mutation in exon 12, found in the first two Bulgarian patients with adult-onset Pompe disease, may be typical for the Balkan population.

